# Transgenic overexpression of γ-cytoplasmic actin protects against eccentric contraction-induced force loss in *mdx *mice

**DOI:** 10.1186/2044-5040-1-32

**Published:** 2011-10-13

**Authors:** Kristen A Baltgalvis, Michele A Jaeger, Daniel P Fitzsimons, Stanley A Thayer, Dawn A Lowe, James M Ervasti

**Affiliations:** 1 Department of Biochemistry, Molecular Biology and Biophysics, University of Minnesota, 6-155 Jackson Hall, 321 Church Street SE, Minneapolis, MN 55455, USA; 2 Department of Cell and Regenerative Biology, University of Wisconsin, 1300 University Avenue, Madison, WI, 53706, USA; 3 Department of Pharmacology, University of Minnesota, 6-120 Jackson Hall, 321 Church Street SE, Minneapolis, MN, 55455, USA; 4 Program in Physical Therapy, Department of Physical Medicine & Rehabilitation University of Minnesota, 420 Delaware Street SE, Minneapolis, MN, 55454, USA

**Keywords:** stretch-activated channels, calcium, skeletal muscle injury, dystrophin, costamere

## Abstract

**Background:**

γ-cytoplasmic (γ-_cyto_) actin levels are elevated in dystrophin-deficient *mdx *mouse skeletal muscle. The purpose of this study was to determine whether further elevation of γ-_cyto _actin levels improve or exacerbate the dystrophic phenotype of *mdx *mice.

**Methods:**

We transgenically overexpressed γ-_cyto _actin, specifically in skeletal muscle of mdx mice (*mdx*-TG), and compared skeletal muscle pathology and force-generating capacity between *mdx *and *mdx*-TG mice at different ages. We investigated the mechanism by which γ-_cyto _actin provides protection from force loss by studying the role of calcium channels and stretch-activated channels in isolated skeletal muscles and muscle fibers. Analysis of variance or independent *t*-tests were used to detect statistical differences between groups.

**Results:**

Levels of γ-_cyto _actin in *mdx*-TG skeletal muscle were elevated 200-fold compared to *mdx *skeletal muscle and incorporated into thin filaments. Overexpression of γ-_cyto _actin had little effect on most parameters of *mdx *muscle pathology. However, γ-_cyto _actin provided statistically significant protection against force loss during eccentric contractions. Store-operated calcium entry across the sarcolemma did not differ between *mdx *fibers compared to wild-type fibers. Additionally, the omission of extracellular calcium or the addition of streptomycin to block stretch-activated channels did not improve the force-generating capacity of isolated extensor digitorum longus muscles from *mdx *mice during eccentric contractions.

**Conclusions:**

The data presented in this study indicate that upregulation of γ-_cyto _actin in dystrophic skeletal muscle can attenuate force loss during eccentric contractions and that the mechanism is independent of activation of stretch-activated channels and the accumulation of extracellular calcium.

## Background

Duchenne muscular dystrophy (DMD) is a severe muscle-wasting disease caused by mutations in the dystrophin gene. Dystrophin localizes primarily to costameres, where it links the cortical actin cytoskeleton to the sarcolemma and the extracellular matrix [1]. It is part of the dystrophin-glycoprotein complex (DGC), a large oligomeric complex of proteins thought primarily to stabilize the sarcolemma during muscle contraction. In the absence of a functional dystrophin protein, myofibers are susceptible to injury, leading to muscle degeneration, inflammation and fibrosis.

Dystrophin binds to filamentous actin with high affinity via two distinct actin-binding domains: an N-terminal tandem calponin homology domain and a region of basic spectrin-like repeats in the middle rod domain [1]. In skeletal muscle, dystrophin forms a mechanically strong link with the cortical actin cytoskeleton, which is composed of γ-cytoplasmic (γ-_cyto_) and β-cytoplasmic actins [2,3]. The tight association between dystrophin and actin has been demonstrated with inside-out peeled sarcolemma from wild-type (WT) myofibers in which γ-_cyto _actin was retained in a riblike costameric pattern but absent from the dystrophin-deficient *mdx *mouse sarcolemma [2]. The importance of dystrophin-actin binding *in vivo *is also evident in transgenic *mdx *mice that express various dystrophin deletion constructs. Despite restoration of DGC members at the sarcolemma, at least one actin-binding domain is essential for partial or complete rescue of the dystrophic phenotype in transgenic *mdx *mice [4-6].

A robust phenotype of the *mdx *mouse is increased susceptibility to injury following eccentric muscle contractions [7,8], a standard procedure to determine the efficacy of a drug or therapy in preclinical trials [9]. Force loss following eccentric contractions depends on the number of contractions, speed of contraction, overall length change, muscle type and age of the mice. Overall force loss in WT mice ranges from 2% to 29%, but force loss can range from 27% to 69% in *mdx *mice [10]. The mechanism of force loss following eccentric contractions in healthy muscle has been investigated. The majority of force loss following eccentric contractions in an *in vivo *rodent model was due to excitation-contraction uncoupling, and about 25% of the force loss can be attributed to physical damage [11]. After the initial bout of injury, the loss of contractile protein accounts for strength loss during 14 to 28 days of recovery. It is unknown whether excitation-contraction uncoupling, physical damage, loss of contractile protein or a combination of different mechanisms explains the rapid force loss in *mdx *mice following eccentric contractions.

Recent studies have suggested that stretch-activated cation channels (calcium or sodium) at the sarcolemma contribute to force loss during eccentric contractions [12-15]. The removal of calcium in the muscle-bathing media or the addition of streptomycin to block stretch-activated channels has been shown to reverse or attenuate force loss during injurious muscle contractions in dystrophic muscle [12,15-17]. Store-operated calcium entry (SOCE) is an important mechanism whereby calcium is brought into muscle cells during sarcoplasmic reticulum (SR) calcium depletion [18]. It has been proposed that SOCE occurs through either (1) an interaction between transient receptor potential C (TRPC) channels and either the ryanodine receptor or inositol trisphosphate receptors or (2) the STIM1/Orai1 pathway [19]. TRP channels are plasma membrane channels that vary in cation selectivity and sensitivity to mechanical stress. While TRP gene transcripts are generally in low abundance in skeletal muscle compared to some other tissues, TRPC3 is the most abundant, with TRP vanilloid 3 (TRPV3), TRPV4, TRPV6, TRP melastatin 3 (TRPM3), TRPM4 and TRPM7 also present [20]. In fact, the overexpression of dominant-negative TRPC3 or TRPV2 channels in muscle ameliorates pathological changes [13,21,22], and TRPC3 overexpression in WT mice is sufficient to induce muscular dystrophy [22]. *mdx *mice that overexpress a dominant-negative TRPV2 channel are also protected from extreme force loss following eccentric muscle contractions [13].

Although γ-_cyto _actin comprises only 1/4, 000th of the actin population in skeletal muscle [23], it is essential for the maintenance of normal muscle function in adults [24]. We previously reported that γ-_cyto _actin levels are elevated fivefold in dystrophin-deficient skeletal muscle [23,25,26] and that muscle-specific ablation of γ-_cyto _actin does not exacerbate the dystrophic condition [26]. As many costameric components are actin-binding proteins, it has been proposed that this increased γ-_cyto _actin expression may contribute to a compensatory remodeling response to reinforce the compromised sarcolemma in *mdx *mice [23]. Understanding the function of increased γ-_cyto _actin in dystrophic muscle may identify a unique pharmacological target for the treatment of muscular dystrophies. The purpose of this study was to determine whether further elevation of γ-_cyto _actin levels improves or exacerbates the dystrophic phenotype of *mdx *mice. We generated a line of transgenic *mdx *mice (*mdx*-TG) that expressed γ-_cyto _actin 200-fold above *mdx *γ-_cyto _actin levels and showed substantial incorporation of γ-_cyto _actin into thin filaments. Interestingly, *mdx*-TG mice showed significantly attenuated force loss during eccentric muscle contractions through a mechanism independent of extracellular calcium and stretch-activated channels.

## Methods

### Generation of transgenic mice

Generation of *Actg1*-TG mice has been previously described [27]. To generate dystrophin-deficient *mdx*-TG mice carrying the *Actg1 *transgene, *Actg1*-TG male mice were crossed with *mdx *females. TG-positive males were subsequently crossed with *mdx *females to generate *mdx*-TG mice. Animals were housed and treated in accordance with the standards set by the Institutional Animal Care and Use Committees at the University of Minnesota and the University of Wisconsin.

### Determination of γ-cytoplasmic actin concentration in skeletal muscle

Known amounts of purified bovine brain actin [27] and SDS extracts from *mdx*-TG gastrocnemius or quadriceps femoris muscles were run side-by-side on a 3% to 12% SDS-polyacrylamide gel and transferred to nitrocellulose membrane. Two γ-_cyto _actin antibodies (monoclonal mouse 2 to 4 and polyclonal affinity-purified rabbit 7577) were used for Western blot analysis, each at a 1:1, 000 dilution. To determine the concentration of actin (in micrograms) in the SDS extracts, a concentration standard curve was derived from the known values of purified bovine γ-_cyto _actin using the densitometry and concentration standards feature in the LI-COR Odyssey Application Software version 2.1.12 (LI-COR Biosciences, Lincoln, NE, USA).

### Skeletal muscle histology and confocal microscopy

Tibialis anterior (TA) and gastrocnemius muscles from WT, *mdx *and *mdx*-TG mice were frozen in melting isopentane and mounted in O.C.T. mounting medium for cryosectioning [27]. Ten-micrometer sections were stained with hematoxylin and eosin. Centrally nucleated fibers (CNFs) were counted on whole sections and expressed as a percentage of the total number of fibers.

Isolation of myofibrils and confocal microscopy were both performed as previously described [27]. Cross-sections of gastrocnemius muscles or myofibrils were stained using the following antibodies: affinity-purified γ-_cyto _actin rabbit 7577 (1:75 dilution; Sigma, St Louis, MO, USA), fast myosin heavy chain clone MY-32 (1:100 dilution; Sigma) and laminin α-2 rat clone 4H8-2 (1:200 dilution; Sigma). All images were obtained using a FluoView FV1000 single-photon confocal microscope equipped with a PlanAPO 60×/1.40 na oil immersion lens objective, a UPlan Fluorite N 1.30 NA 40× oil immersion lens objective and a UPLSAPO 0.40 NA 10× lens objective (all from Olympus America Inc, Melville, NY, USA). Images were collected with Olympus FluoView FV1000 version 1.7b software and assembled and processed using ImageJ software (National Institutes of Health, Bethesda, MD, USA) and Adobe Photoshop version 8.0 software (Adobe Systems Inc, San Jose, CA, USA).

### Serum creatine kinase analysis

Serum was collected and stored at -80°C until analysis. Ten microliters of serum were applied to a VITROS DT60 II MicroSlide (Ortho-Clinical Diagnostics, Inc, Rochester, NY, USA) and creatine kinase (CK) activity measured using a Kodak Ektachem DT60 Analyzer (Eastman Kodak Co, Rochester, NY, USA) and a VITROS DTSC II Module (Ortho-Clinical Diagnostics, Inc).

### *Ex vivo *contractility measurements and eccentric injury protocol

The extensor digitorum longus (EDL) muscles were assessed for contractile function and susceptibility to eccentric contraction-induced injury as previously described [26,28]. Age-matched male *mdx *and *mdx*-TG mice (*n *= 5 or 6 per group) were anesthetized with sodium pentobarbital (100 mg/kg body weight). Their muscles were dissected and mounted onto a dual-mode muscle lever system (model 300B-LR Dual-Mode Lever Arm; Aurora Scientific Inc, Aurora, ON, Canada) with 4-0 suture in a 1.5-ml bath assembly filled with Krebs-Ringer bicarbonate buffer that was maintained at 25°C and perfused with 95% O_2_. The muscles were adjusted to their anatomic optimal length (L_o_) based on resting tension of 0.4 g, and then muscle length was measured. The muscles remained quiescent in the bath for ten minutes. Passive stiffness, twitch force (P_t_), tetanic force (P_o_) and active stiffness were measured prior to initiating the eccentric injury protocol. First, passive stiffness of the muscle was determined by stretching the muscle sinusoidally from 97.5% L_o _to 102.5% L_o _at 0.5 Hz while measuring the resulting force [29,30]. Two twitches (P_t_) separated by 30-second intervals were elicited, and the resulting force was measured. Maximal isometric tetanic contractions (P_o_) were obtained by stimulating muscles for 400 ms at 180 Hz and 150 V (Grass S48 Square Pulse Stimulator; Grass Technologies, West Warwick, RI, USA) delivered through a SIU-V stimulus isolation unit (Grass Technologies). Two minutes later a second tetanic isometric contraction was elicited, and at peak force a sinusoidal length oscillation of 0.01% L_o _at 500 Hz was imposed to determine active stiffness, an indirect marker of strong-binding myosin [29,30]. Two minutes after that an injury protocol consisting of five to ten eccentric contractions was begun. For these contractions, muscles were passively shortened from L_o _to 0.95 L_o _over 3 seconds, stimulated tetanically for 200 ms as the muscle lengthened to 1.05 L_o _at 0.5 L_o_/second and then passively returned to L_o_. Each eccentric contraction was separated by three minutes of rest. The degree of injury was calculated as the percentage change in eccentric force production from the first to the last eccentric contraction. Specific force was calculated by dividing isometric force by the anatomical cross-sectional area of the muscle. The cross-sectional area of the muscle was calculated by dividing the mass of the muscle by the product of fiber length (L_f_) (L_o _× 0.44) and the density of muscle (1.06 g/mm^3^). Contractility and injury protocols were followed to study both the right and left sides and averaged as a single data point for each mouse when comparing *mdx *and *mdx*-TG mice.

To determine the effects of extracellular calcium and stretch-activated channels on the susceptibility to eccentric contraction-induced injury, three different conditions were tested, all using male *mdx *mice, with the contralateral muscle from the same mouse used as a control (*n *= 3 to 5 per group). In the first condition, Krebs-Ringer bicarbonate buffer was made without CaCl_2 _but replaced with the same molar ratio of MgCl_2 _(that is, calcium-free). In the second condition, 1 mM ethylene glycol tetraacetic acid (EGTA) was added to the calcium-free buffer. Pilot work revealed that EGTA blunted isometric force production, so buffer containing 1 mM EGTA was introduced only after the preinjury contractile measurements were completed. The muscle remained quiescent for ten minutes after the addition of the calcium-free buffer with EGTA before the eccentric contraction protocol was begun. In the third condition, 2 mM streptomycin was added to Krebs-Ringer bicarbonate buffer for the entire protocol. Pilot work revealed a greater effect on postinjury isometric force production with the 2 mM concentration instead of the previously reported 0.2 mM [12].

### Flexor digitorum brevis myofiber isolation

Flexor digitorum brevis (FDB) fibers were isolated from WT and *mdx *mice as previously described [31-33]. The right and left FDB muscles were dissected from WT, *mdx *and *mdx*-TG mice and placed in 5 ml of sterile physiological rodent saline (138 mM NaCl, 2.7 mM KCl, 1.8 mM CaCl_2_, 1.06 mM MgCl_2_, 12.4 mM 4-(2-hydroxyethyl)-1-piperazineethanesulfonic acid (HEPES), 5.6 mM glucose, pH 7.3). The muscles were then transferred to a Petri dish containing 2 ml of media (DMEM, 10% fetal bovine serum, and 0.5% penicillin/streptomycin) containing 2 mg/ml collagenase. Following this step, they were incubated at 37°C (5% CO_2_) for three hours and then gently triturated in fresh media and allowed to settle at 1 × *g*. All but 1 ml of the media was aspirated, and the fiber pellet was gently resuspended. Next the fibers were placed into six 35-mm Petri dishes, each containing a glass coverslip coated with 1 μg of laminin supplemented with 2 ml of media. The fibers were allowed to adhere to the coverslips overnight at 37°C. They were analyzed for SOCE within 24 to 48 hours after dissection.

### Store-operated calcium entry

SOCE was measured in FDB fibers as previously described [22,34]. Coverslips containing adhered FDB fibers were briefly washed in physiological saline solution (PSS) (130 mM NaCl, 5.6 mM KCl, 1 mM MgCl_2_, 1.7 mM CaCl_2_, 11 mM glucose, 10 mM HEPES, pH 7.4). Indo-1 AM (Invitrogen, Carlsbad, CA, USA) was mixed 1:1 with Pluronic F-127 solution (Invitrogen) and added to myofibers at a final concentration of 1 μM. Myofibers were loaded with dye for 30 minutes at 37°C and washed with PSS for 15 minutes at 37°C. Instrumentation and recording of the calcium signal was performed as previously described [35]. Myofibers were perfused with PSS at a rate of 1 to 2 ml/minute for two minutes before being moved to calcium-free PSS. The calcium-free PSS did not contain CaCl_2_, had a MgCl_2 _concentration of 2 mM, contained 1 mM EGTA and contained 30 μM cyclopiazonic acid (CPA) to block SR calcium-ATPase. Calcium-free PSS was perfused for four to eight minutes before 5 mM caffeine was added to release calcium from the SR. After the indo-1 ratio had returned to baseline (8 to 12 minutes), the muscle was perfused with PSS and SOCE was calculated as the difference in the minimum and maximum indo-1 ratios averaged over a 30-second period. During the recording of the indo-1 signal, 50 μM *N*-benzyl-*p*-toluene sulfonamide (BTS) was added to all solutions to prevent myofiber contractions.

### Data analysis

Two-way analysis of variance (ANOVA) was used to assess differences in CNFs and serum CK with mouse strain (WT vs *mdx *vs *mdx*-TG) and age as the factors. One-way ANOVA was used to compare intracellular calcium concentration ([Ca^2+^]_i_) between WT, *mdx *and *mdx*-TG mice. *Post hoc *analyses were performed using the Holm-Sidak test. *t*-tests were used to analyze differences between all other variables. Data are reported as means ± SEM. Significance was set at *P *< 0.05.

## Results

**Figure 1:**
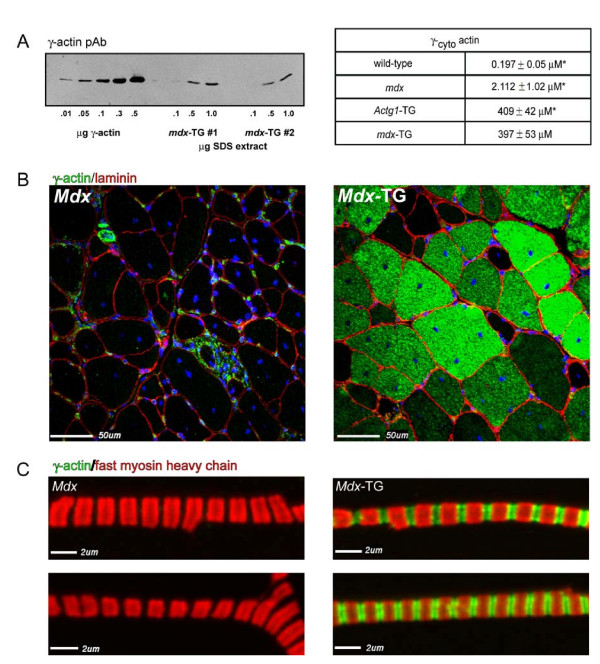
**γ-cytoplasmic actin expression in skeletal muscle and incorporation into thin filaments**. **(A) **Quantitative Western blot analysis of γ-cytoplasmic (γ-_cyto_) actin levels in SDS extracts of *mdx*-TG murine skeletal muscle. Values are means +/- SEM. *Previously published values [23,27]. **(B) **γ-_cyto _actin expression in *mdx *and *mdx*-TG gastrocnemius muscle cross-sections. **(C) **γ-_cyto _actin expression in thin filaments. Isolated myofibrils were costained with antibodies against γ-_cyto _actin and fast myosin heavy chain.

To examine the role of elevated γ-_cyto _actin in dystrophin-deficient muscle, *Actg1*-TG male mice were crossed with *mdx *female mice. Levels of γ-_cyto _actin in *mdx*-TG skeletal muscle SDS extracts were analyzed alongside a purified γ-_cyto _actin calibration curve on SDS-PAGE gels and blotted with γ-_cyto _actin-specific antibodies. Mean (± SEM) *mdx*-TG γ-_cyto _actin levels in muscle were 397 ± 53 μM, which is approximately 200-fold that in *mdx *skeletal muscle and similar to that in skeletal muscles of *Actg1*-TG mice (Figure 1A).

To determine the localization of γ-_cyto _in *mdx*-TG skeletal muscle, cross-sections were stained with an affinity-purified γ-_cyto _actin antibody. Capillaries, blood vessels, nerves and immune cells stained brightly in *mdx *skeletal muscle (Figure 1B). In addition, the internal structure of *mdx*-TG muscle showed intense γ-_cyto _actin localization as previously described [27] for *Actg1*-TG mice (Figure 1B). Internal staining of γ-_cyto _actin was prominent in the majority of fibers. Staining of isolated *mdx*-TG myofibrils showed γ-_cyto _actin incorporation into the thin filament that was absent in *mdx *myofibrils (Figure 1C). Colocalization of γ-_cyto _actin with fast myosin heavy chain is consistent with reports that the human α-skeletal actin promoter used to drive transgene expression is preferentially active in fast-twitch muscle fibers [27,36].

**Figure 2:**
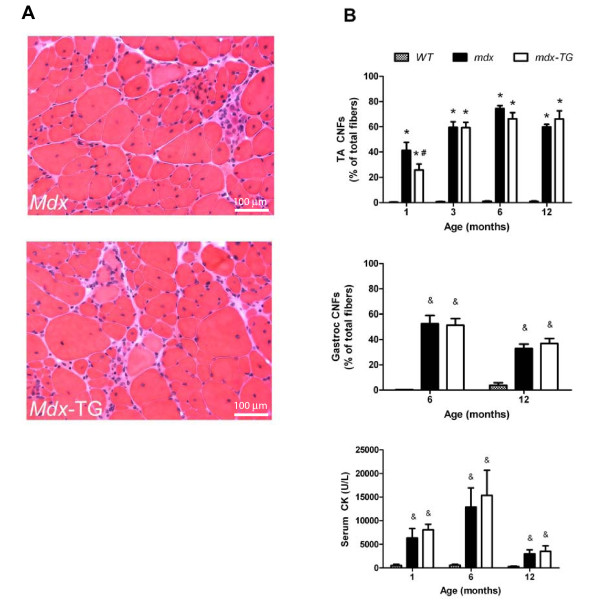
**Overexpression of γ-cytoplasmic actin does not alter muscle pathology or serum creatine kinase**. **(A) **Representative muscle tissue cross-sections from *mdx *and *mdx*-transgenic (*mdx*-TG) mice (hematoxylin and eosin stain). **(B) **Centrally nucleated fibers (CNFs) in tibialis anterior (TA) and gastrocnemius muscles and serum creatine kinase (CK) levels from wild-type (WT), *mdx *and *mdx*-TG mice at 4 to 52 weeks of age. *Statistically significant difference from WT. #Statistically significant difference from *mdx*. &Statistically significant difference from WT (main effect of strain).

To determine whether elevated levels of γ-_cyto _actin were beneficial or detrimental to dystrophin-deficient skeletal muscle, several well-established parameters of dystrophic pathology were examined. Hematoxylin and eosin-stained skeletal muscle cross-sections from *mdx *and *mdx*-TG mice were indistinguishable, showing characteristic fiber size variations, necrotic cells, fibrosis and mononuclear cell infiltration (Figure 2A). In addition, *mdx *mice exhibited high levels of CNFs and elevated serum CK. CNF levels were quantified in gastrocnemius and TA muscles at various ages (Figure 2B). As expected, *mdx *and *mdx*-TG mice had greater CNFs in the TA muscle (*P *< 0.001) and the gastrocnemius muscle (*P *< 0.001) compared to WT mice. In the TA muscle, there was a significant interaction between mouse group and age (*P *< 0.001). *mdx*-TG mice had 37% fewer CNFs in the TA muscle at one month of age compared to *mdx *mice, but there were no differences in CNFs between *mdx *and *mdx*-TG mice at any other ages for the TA muscle (*P *> 0.155) or the gastrocnemius muscle (*P *= 0.793). Serum CK levels were elevated in dystrophic compared to WT mice (*P *≤ 0.013) but were not different between *mdx *and *mdx*-TG mice, regardless of age (*P *= 0.463) (Figure 2B).

**Figure 3:**
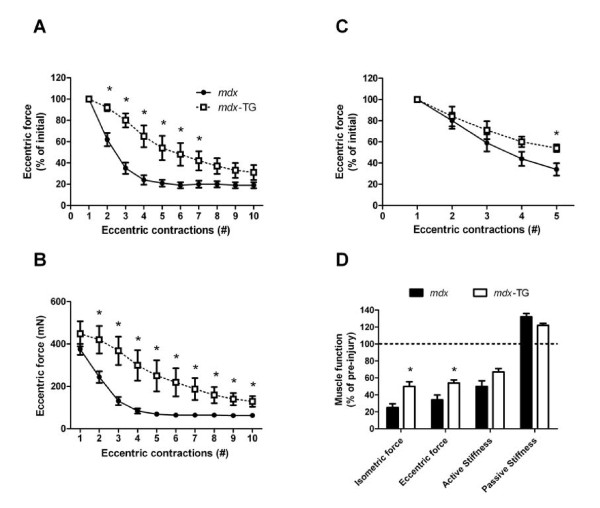
**γ-cytoplasmic actin overexpression in *mdx *mice attenuates force loss during eccentric contractions**. Eccentric contractions were performed *ex vivo *in isolated extensor digitorum longus (EDL) muscles from *mdx *and *mdx*-TG mice at one and four months of age. **(A) **Force loss expressed as a percentage of initial eccentric force in four-month-old mice. **(B) **Eccentric force generation at each contraction in four-month-old mice. **(C) **Force loss in one-month-old mice. **(D) **Postinjury functional analyses of one-month-old mice. Data are means ± SEM. *Statistically significant difference from *mdx *mice.

We previously demonstrated that *Actg1*-TG mice exhibit normal *ex vivo *and *in vivo *muscle performance, despite replacing 40% of the contractile α-_skeletal _actin population with γ-_cyto _actin [27]. In EDL muscles from *mdx*-TG mice, passive stiffness was 27% less and wet mass was 13% less than those from the EDL muscles of *mdx *mice. There were no differences in any other parameters of isometric contractility including P_t_, P_o_, specific force, rate of contraction, rate of relaxation, time to P_t _or one-half relaxation time when γ-_cyto _actin was overexpressed in dystrophic muscle (*P *≥ 0.140) (Table 1). Most interestingly, when isolated EDL muscles from *mdx *and *mdx*-TG mice were subjected to a series of ten eccentric contractions, force loss was attenuated in *mdx*-TG mice during contractions 2 through 7 (*P *≤ 0.044) (Figure 3A). The reduced susceptibility to injury in the *mdx*-TG mice was not due to lower force production, because *mdx*-TG mice produced 72% to 263% more eccentric force during eccentric contractions 2 through 10 compared to *mdx *mice (*P *≤ 0.041) (Figure 3B). We followed up this experiment by testing susceptibility to injury in younger *mdx *and *mdx*-TG mice and also by decreasing the number of eccentric contractions. One-month-old *mdx*-TG mice also demonstrated attenuated force loss during eccentric contractions (Figure 3C), although the phenotype was not quite as robust. Postinjury isometric contractile measurements in one-month-old *mdx*-TG mice showed less isometric force loss and less eccentric force loss compared to *mdx *mice (*P *≤ 0.018) (Figure 3D). There were also trends toward reduced passive stiffness (*P *= 0.071) and active stiffness (*P *= 0.070) following injury in *mdx*-TG mice compared to *mdx *mice.

**Table 1:** Isometric contractile properties of extensor digitorum longus muscles from four-month-old *mdx *and *mdx*-TG mice

Contractile properties	*mdx *(*n *= 6)	*mdx*-TG (*n *= 6)	*P *value
EDL mass (mg)	15.8 ± 0.8	13.7 ± 0.5	0.043*
L_o _(mm)	11.9 ± 0.2	12.2 ± 0.1	0.205
CSA (cm^2^)	0.028 ± 0.001	0.024 ± 0.001	0.009*
Passive stiffness (N/m)	19.6 ± 1.6	14.2 ± 0.9	0.018*
Active stiffness (N/m)	714.8 ± 40.1	738.7 ± 98.3	0.826
P_t _(mN)	59.6 ± 5.6	65.7 ± 8.1	0.593
TPT (ms)	17.9 ± 0.4	18.3 ± 0.4	0.556
RT_1/2 _(ms)	21.4 ± 1.3	19.1 ± 0.7	0.161
Specific P_t _(N/cm^2^)	2.1 ± 0.2	2.8 ± 0.4	0.153
P_o _(mN)	264.0 ± 21.5	288.6 ± 39.1	0.593
+d*P*/d*t *(N/s)	7.15 ± 0.63	7.68 ± 0.95	0.648
-d*P*/d*t *(N/s)	10.9 ± 1.2	13.0 ± 2.4	0.458
Specific P_o _(N/cm^2^)	9.1 ± 0.6	12.2 ± 1.8	0.140

Values are means ± SEM. CSA = cross-sectional area; +d*P*/d*t *= maximum rate of tetanic force development; -d*P*/d*t *= maximum rate of relaxation; EDL = extensor digitorum longus; L_o _= optimal length; P_o _= peak tetanic force; P_t _= peak twitch force; RT_1/2 _= one-half relaxation time; TG = transgenic; TPT = time to peak twitch force. *Statistically significant difference from *mdx*.

**Figure 4:**
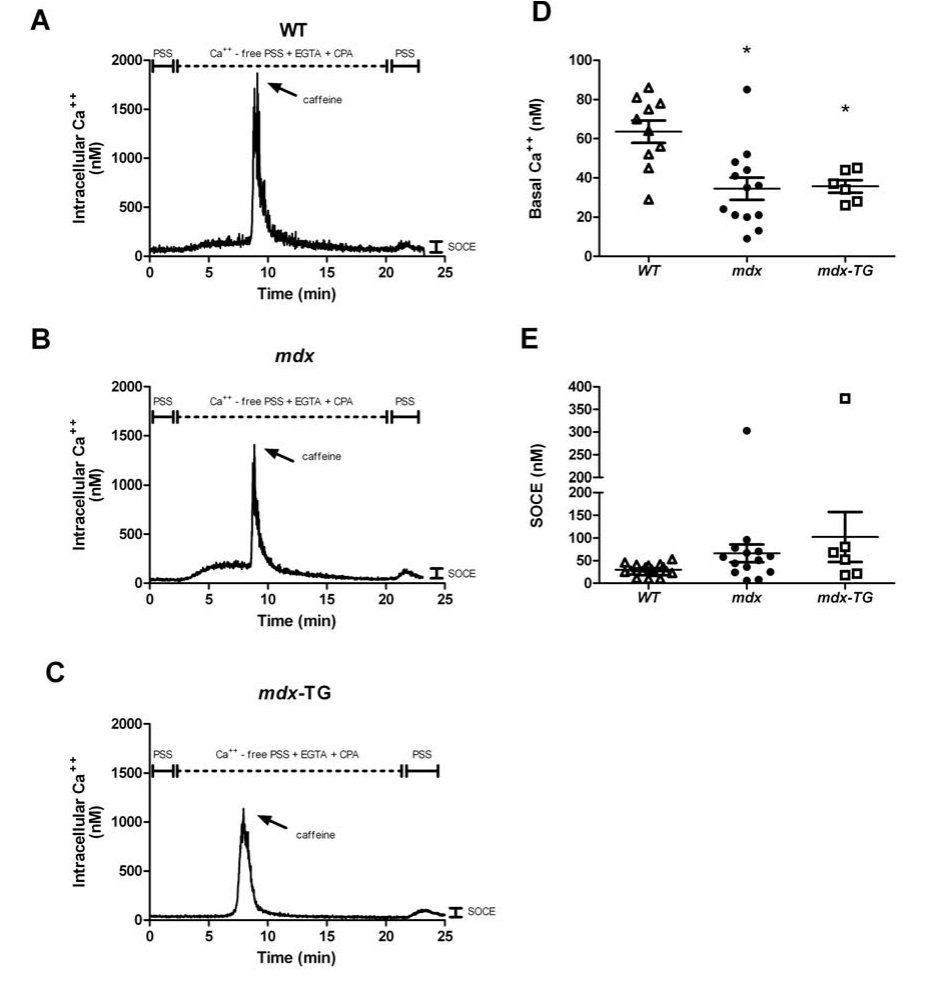
**Store-operated calcium entry in flexor digitorum brevis myofibers**. Representative calcium tracings in wild-type (WT) **(A)**, *mdx ***(B) **and *mdx*-transgenic (*mdx*-TG) **(C) **flexor digitorum brevis (FDB) fibers following sarcoplasmic reticulum (SR) depletion and add-back of calcium. **(D) **Basal intracellular calcium concentration ([Ca^2+^]_i_) in WT, *mdx *and *mdx*-TG FDB fibers. **(E) **[Ca^2+^]_i _during store-operated calcium entry (SOCE). Bars represent means ± SEM. *Statistically significant difference from WT mice.

The protection afforded by γ-_cyto _actin could be manifested through many mechanisms. We were intrigued by how similar our data were to those reported by Zanou *et al*. [13], who demonstrated that *mdx *mice with high levels of a dominant-negative TRPV2 channel were protected from contraction-induced injury, despite having normal isometric and eccentric force generation and smaller EDL muscles. These results resemble ours from mice overexpressing γ-_cyto _actin. Therefore, we designed experiments to test the hypothesis that overexpression of γ-_cyto _actin reduces the function of stretch-activated channels to prevent abnormal influx of calcium during eccentric contractions. Since there has been some controversy whether SOCE and TRP channel functions are perturbed in *mdx *muscle, we first examined SOCE in isolated FDB fibers from WT mice (Figure 4A), *mdx *mice (Figure 4B) and *mdx*-TG mice (Figure 4C). Muscle contraction was blocked by the addition of BTS. Basal [Ca^2+^]_i _was significantly less in *mdx *fibers (35 ± 6 nM) and *mdx*-TG fibers (36 ± 3 nM) compared to WT fibers (62 ± 5 nM; *P *= 0.002) (Figure 4D). The SR was depleted of calcium by application of CPA and caffeine in calcium-free PSS. SOCE was evoked by returning calcium to the bath (Figure 4E). In WT fibers, SOCE produced a minor detectable [Ca^2+^]_i _increase (30 ± 3 nM). This response was more variable in *mdx *fibers (66 ± 20 nM) and *mdx*-TG fibers (102 ± 55 nM), but these mean values were not statistically different from WT fibers (*P *= 0.085). The peak [Ca^2+^]_i _following the replacement of the perfusate with calcium was not different between WT mice (96 ± 7 nM), *mdx *mice (113 ± 20 nM) and *mdx*-TG mice (134 ± 54 nM; *P *= 0.968).

**Figure 5:**
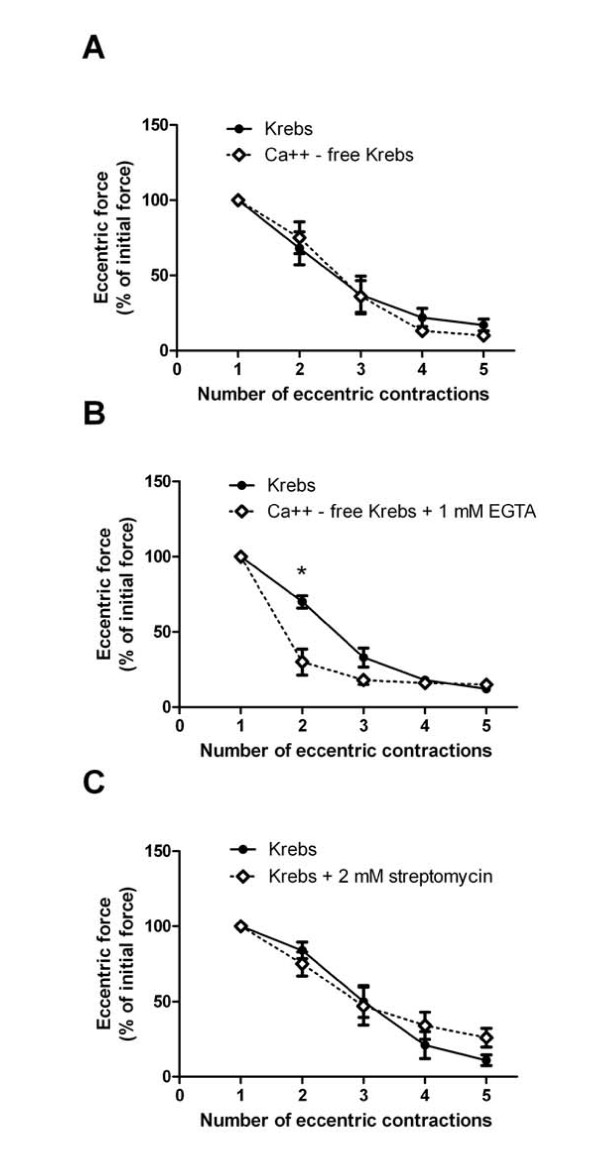
**Removal of extracellular calcium or addition of streptomycin does not affect immediate force loss during eccentric muscle contractions in *mdx *mice**. **(A) **Eccentric contractions performed in calcium-free buffer (CaCl_2 _replaced with MgCl_2_). **(B) **Eccentric contractions performed in calcium-free buffer with the addition of 1 mM ethylene glycol tetraacetic acid (EGTA). **(C) **Eccentric contractions performed in normal buffer with the addition of 2 mM streptomycin. Data are means ± SEM. *Statistically significant difference from Krebs-Ringer bicarbonate buffer.

This result prompted us to examine the effect of extracellular calcium and stretch-activated channels on force loss during eccentric contractions in intact *mdx *EDL muscles. First, we substituted MgCl_2 _for CaCl_2 _in the bath during contractions, which did not attenuate force loss (Figure 5A). Second, we used the same calcium-free buffer and added 1 mM EGTA to chelate any contaminating calcium. Instead of preventing force loss, muscles bathed in a calcium-free buffer with EGTA lost more force at the second eccentric contraction (*P *= 0.013) (Figure 5B). In the third experiment, 2 mM streptomycin was added to the Krebs-Ringer bicarbonate buffer to block stretch-activated channels. Streptomycin did not affect eccentric force loss at any eccentric contraction (*P *≥ 0.088) (Figure 5C).

**Figure 6:**
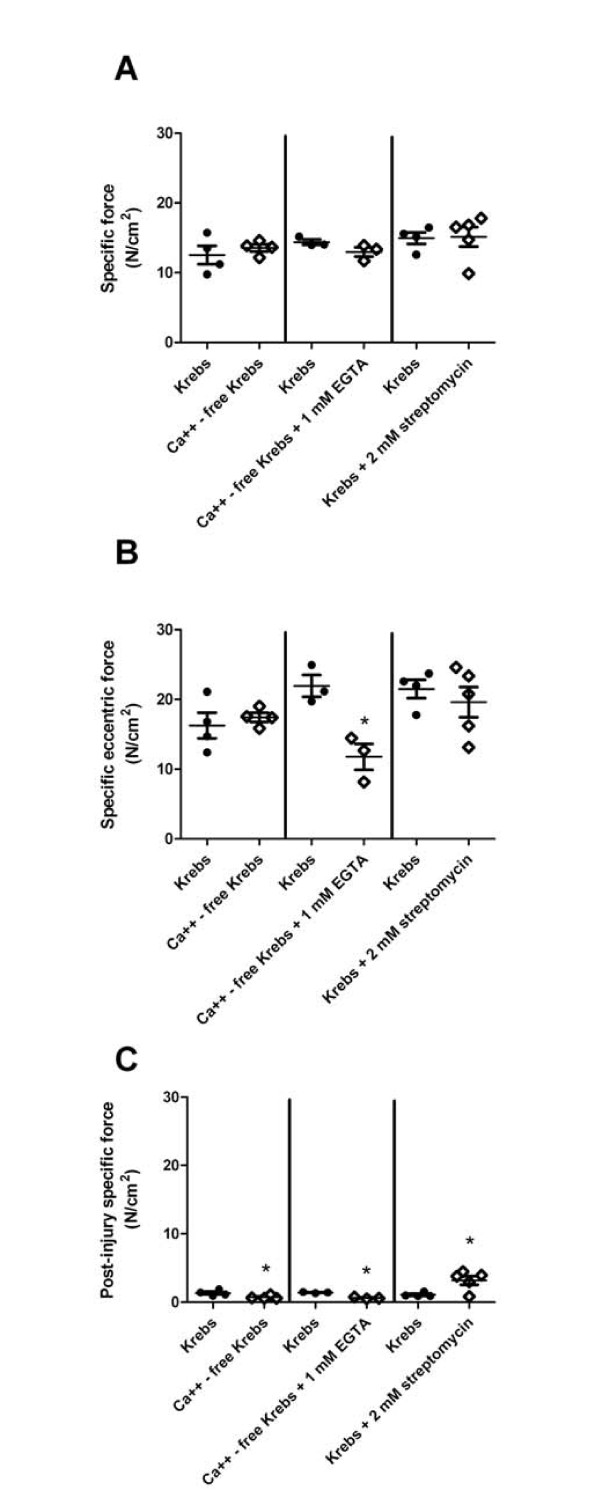
**Isometric specific force after eccentric contractions in *mdx *mice is improved with streptomycin, but not in a calcium-free buffer**. **(A) **Isometric specific force before injury. **(B) **Eccentric specific force of first eccentric contraction. **(C) **Isometric specific force measured three minutes after eccentric contractions. Data are means ± SEM. *Statistically significant difference from Krebs-Ringer bicarbonate buffer.

It is important to consider whether the removal of calcium or the addition of EGTA or streptomycin affected isometric or eccentric force production, because such effects would consequently affect force loss. Specific isometric force production was unaffected by the removal of calcium or the addition of streptomycin (*P *≥ 0.139) (Figure 6A). Pilot work showed that EGTA affected isometric force, so the calcium-free buffer containing EGTA was added to the bath after we took isometric measurements in normal Krebs-Ringer bicarbonate buffer. However, specific eccentric force production of muscles bathed in a calcium-free solution containing EGTA was approximately 50% less than that in normal Krebs-Ringer bicarbonate buffer (*P *= 0.014) (Figure 6B). Interestingly, although eccentric force loss by the fifth contraction was not altered in any of the different buffers, isometric force production measured three minutes after the last eccentric contraction was either 44% or 53% of that of control muscles in muscles bathed in a calcium-free buffer with or without EGTA, respectively. Conversely, muscles bathed with streptomycin had greater isometric force nearly threefold that of control muscles.

## Discussion

We are just beginning to understand the roles of cytoplasmic actin isoforms in skeletal muscle. Nonmuscle γ-_cyto _actin plays a vital role in muscle maintenance, as its ablation results in the accumulation of CNFs and hindlimb contractures [24]. In dystrophic muscle, γ-_cyto _actin levels are elevated five- to tenfold compared to WT muscle, compensating for a weakened DGC and sarcolemma [23,25]. When transgenically overexpressed 2, 000-fold (about 400 μM) in WT mice, γ-_cyto _actin incorporated into myofibrils but had no effect on isometric force generation, muscle fiber size or the incidence of CNFs [27]. Similarly, we found that γ-_cyto _actin overexpression in dystrophic muscle also did not affect pathology, isometric force production or disease progression.

Our most interesting finding is that γ-_cyto _actin overexpression significantly decreased the immediate force loss caused by eccentric contractions in *mdx *muscle. This protection was apparent at two different ages of mice and was also associated with greater isometric force generation at the end of the eccentric contractions. Thus the protection afforded by γ-_cyto _actin overexpression was not due to weakened muscles, as isometric force production before the injury protocol was similar between *mdx *and *mdx*-TG mice and four-month-old *mdx*-TG mice actually generated greater eccentric force in seven of ten eccentric contractions compared to *mdx *mice. After eccentric contractions, EDL muscle mass was less in *mdx*-TG mice than in *mdx *mice, and there was a trend toward a greater passive stiffness increase in *mdx *mice than in *mdx*-TG mice, possibly suggesting reduced fluid accumulation as a consequence of muscle damage. These results differ from our findings in WT mice that *Actg1*-TG mice show a similar susceptibility to injury following eccentric contractions. It is possible that γ-_cyto _actin's protective effect in dystrophic muscle corrects a mechanism associated with force loss in dystrophic muscle that is not present in WT muscle.

The mechanism of force loss following eccentric contractions in dystrophic muscle is not understood but is likely different from that in healthy muscle. For example, in healthy muscle, excitation-contraction uncoupling has been demonstrated to account for about 75% of the deficit in muscle strength following eccentric contractions *in vivo*, with physical damage accounting for only approximately 25% [11]. It has been postulated that extracellular calcium plays an important role in activating calpains to cause protein degradation. Furthermore, following excessive calcium entry into dystrophic muscle, the mitochondria act as a sink to buffer elevated calcium levels and consequently increase the production of reactive oxygen species [37-39]. Because the actin cytoskeleton can affect SOCE and interact with TRP channels [40], we hypothesized that γ-_cyto _actin's protective effect is due to its ability to attenuate extracellular calcium entry during eccentric contractions. Although *mdx *and *mdx*-TG mice appeared to have a slightly greater SOCE response than WT mice, we were unable to detect a difference between the three genotypes. In fact, the peak [Ca^2+^]_i _was not different between the genotypes, suggesting that any potential differences in SOCE were due to differences in basal [Ca^2+^]_i_. Our data differ from those reported by others who also used a calcium dye with other dystrophic models, such as *mdx^5Cv ^*and sarcoglycan δ-null mice [22,34]. The *mdx^5Cv ^*mouse has fewer revertant fibers and an overall phenotype that is more severe than the *mdx *mouse [41]. It is quite possible that calcium influx during store depletion is a phenotype seen in some mouse models of muscular dystrophy, but not in the *mdx *mouse.

Since we were not able to detect a difference in SOCE in immobilized FDB fibers, we performed more physiologically relevant experiments by modifying extracellular calcium or adding streptomycin to block stretch-activated channels to the muscle bath during *ex vivo *eccentric muscle contractions of the EDL muscle, similarly to previous work [12,14,15]. We found that removal of CaCl_2 _or the blockade of stretch-activated channels in the bathing media did not have an effect on immediate force loss during eccentric contractions. Our results are different from those previously published [12,13,15-17], probably because of the muscles studied (EDL vs FDB fiber or lumbrical muscle), differences in protocol (length change and rest time between contractions), length of treatment (acute vs chronic manipulation of genes) and calculation of force loss (eccentric vs isometric and timing of measurement).

The most commonly used muscle for the assessment of eccentric contraction-induced injury in *mdx *mice is the EDL [10], which we used in the current study. One previous study assessed the role of extracellular calcium and stretch-activated channel blockers during injurious muscle contractions in the EDL muscle [12], whereas all others have used FDB fibers or the lumbrical muscles [14-17]. It appears that isolated muscle fibers [14,15] demonstrate a more robust response than intact muscles to stretch-activated channel blockers [12]. Other studies supporting a role for extracellular calcium in force loss have been performed in the lumbrical muscle, using isometric contractions instead of eccentric contractions [16,17].

Another difference between this and previous studies are the conditions of the eccentric injury protocol. Physiological length changes in muscle in a previous study were 20% or less [42]. Researchers in other studies who have shown positive results by removing extracellular calcium or blocking stretch-activated receptors used length changes of 30% to 40% of L_o _[12,14,15]. In the current study, we were able to use a physiological length change (L_o _± 5%) and still induce a significant amount of force loss. It is possible that a nonphysiological length change (more than 20% L_o_) may stimulate a different mechanism responsible for force loss that is not apparent when the length change is relatively minor. The number of contractions and the rest time between contractions may also be important. The muscles in our study were rested for three minutes between contractions to prevent fatigue. Investigators in other studies have used rest periods ranging from 4 to 60 seconds between contractions. These short rest times can cause fatigue, which affects the amount of injury induced and complicates interpretation. With our three-minute rest period between contractions, we did not see any force loss during isometric or concentric contractions (data not shown).

Recent studies have suggested that TRP channels contribute to the dystrophic phenotype and contraction-induced injury [13,21,22]. Dystrophic mice harboring a dominant-negative TRPV2 or TRPC3 channel transgene show improved CK, fiber size variability, CNFs and susceptibility to contraction-induced injury [13,21,22]. In our study, acute blockage of stretch-activated channels with 2 mM streptomycin did not attenuate eccentric force loss. Mice that chronically overexpress a dominant-negative TRPV2 channel show improvement in CK, CNFs and contraction-induced injury [13,21]. Perhaps chronic blockade of TRP channels leads to a healthier muscle, which indirectly results in protection from eccentric contractions.

It is also imperative to describe how force loss is calculated after eccentric contractions. Herein we report both eccentric force loss and isometric force loss. Although zero extracellular calcium or the addition of streptomycin had no effect on overall eccentric force loss, isometric force loss measured three minutes after the last eccentric contraction was significantly less in the zero calcium buffer but about threefold higher when treated with streptomycin. The reason for these disparate results may lie in the timing of the force measurements after injury. Whitehead *et al*. [12] did not report differences in eccentric force loss immediately after injury, but rather differences in isometric force loss 30 minutes after the last eccentric contraction when bathing EDL muscles in 0.2 mM streptomycin, which is similar to our finding. During that time, isometric force recovered from less than 10% of preinjury force to 34% to 44% of preinjury torque. We suggest that blocking stretch-activated channels enhances the recovery of muscle following eccentric contractions. Since some stretch-activated channels are nonselective for cations and zero extracellular calcium actually resulted in reduced isometric force (with or without EGTA), the effect of streptomycin on the recovery of force may be independent of calcium.

## Conclusions

Our results provide new insight into the mechanism of contraction-induced injury in dystrophic muscle. First, γ-_cyto _actin overexpression can protect muscles from eccentric contraction-induced injury independently of any detectable improvement in dystrophic muscle pathology or disease phenotype. Second, γ-_cyto _actin overexpression appears to act through a mechanism independent of extracellular calcium or stretch-activated channels. Our data also suggest that dysfunction of stretch-activated cation channels is not involved in the immediate force loss following eccentric contractions in dystrophic muscle. Future studies will aim to determine the biochemical and biophysical properties of thin filaments, myofibrils and myofibers composed of different actin isoforms to gain understanding of how altered actin isoform composition translates into altered muscle function.

## Abbreviations

DGC: dystrophin-glycoprotein complex; DMD: Duchenne muscular dystrophy; DMEM: Dulbecco's modified Eagle's medium; EDL: extensor digitorum longus; FDB: flexor digitorum brevis; SOCE: store-operated calcium entry; SR: sarcoplasmic reticulum; STIM1: stromal interacting molecule 1; TRP: transient receptor potential.

## Competing interests

The authors declare that they have no competing interests.

## Authors' contributions

KAB carried out the muscle physiology and calcium experiments, performed all statistical analyses and drafted the manuscript. MAJ bred and characterized the transgenic mice, performed immunofluorescence analysis and contributed to the writing of the manuscript. DPF collected muscle physiological data. SAT contributed to the design, acquisition and interpretation of the calcium experiments. DAL contributed to the design, acquisition and interpretation of the muscle physiological experiments. JME conceived the study and contributed to the writing of the manuscript. All authors participated in revising the manuscript for intellectual content and approved the final manuscript.
